# Markedly elevated blood concentrations of everolimus during concomitant posaconazole therapy in a liver transplant recipient: a case report

**DOI:** 10.1186/s40780-026-00610-1

**Published:** 2026-07-15

**Authors:** Machiko Hirai, Keisuke Umemura, Yoshiki Katada, Yusuke Kojima, Hiroki Ishimura, Yurie Katsube, Daiki Hira, Shoichi Kageyama, Masahiro Tsuda, Shunsaku Nakagawa, Etsuro Hatano, Tomohiro Terada

**Affiliations:** 1 Department of Clinical Pharmacology and Therapeutics, Kyoto University Hospital, 54 Shogoin-Kawahara-cho, Sakyo-ku, Kyoto, 606-8507 Japan; 2 Division of Hepato-Biliary Pancreatic Surgery, Department of Surgery, Graduate School of Medicine, Kyoto University, 54 Shogoin-Kawahara-cho, Sakyo-ku, Kyoto, 606-8507 Japan; 3 Department of Pharmacy, University of Fukui Hospital, 23-3 Matsuoka- Shimoaizuki, Eiheiji-Cho, Yoshida-Gun, Fukui, 910-1193 Japan

**Keywords:** Everolimus, Posaconazole, Tacrolimus, Drug–drug interaction, Liver transplantation, CYP3A

## Abstract

**Background:**

Everolimus (EVL) is widely used as an immunosuppressive agent in combination with calcineurin inhibitors, including tacrolimus (TAC), after liver transplantation. Posaconazole (PSCZ) is a triazole antifungal agent used for the treatment and prophylaxis of invasive fungal infections. Because PSCZ is a potent inhibitor of cytochrome P450 (CYP) 3A4, clinically significant drug–drug interactions with CYP3A substrates may occur. Although both EVL and TAC are primarily metabolized by CYP3A4, quantitative clinical data regarding the relationship between EVL and PSCZ remain limited.

**Case presentation:**

We report the case of a living-donor liver transplant patient receiving EVL and TAC who was treated with PSCZ for suspected allergic bronchopulmonary mycosis. During PSCZ therapy, the concentration/dose (C/D) ratios of EVL and TAC increased by approximately 15-fold and 6-fold, respectively. The PSCZ trough concentration reached 8.8 µg/mL, and the serum potassium level decreased 2.5 mmol/L. Both the magnitude of increase in TAC exposure and increase in EVL exposure were more pronounced than those reported previously. Excessive PSCZ exposure may have augmented CYP3A inhibition, and the greater dependence of EVL metabolism on CYP3A compared to TAC likely contributed to the marked elevation in EVL exposure.

**Conclusions:**

This case suggests that the drug–drug interaction between EVL and PSCZ may be more pronounced than that between TAC and PSCZ in certain cases with high PSCZ concentrations. When PSCZ is co-administered with EVL, close monitoring of the EVL concentration is recommended. Therapeutic drug monitoring of PSCZ may also be considered in selected cases to optimize safety and avoid excessive exposure, particularly when adverse events are suspected or clinically significant drug–drug interactions are anticipated.

## Background

Everolimus (EVL) is a rapamycin derivative that inhibits the mammalian target of rapamycin (mTOR). EVL is used as an immunosuppressant in combination with calcineurin inhibitors (CNIs), including tacrolimus (TAC) and cyclosporine, in liver transplantation recipients [1]. Because liver transplant recipients require long-term immunosuppressive treatment, they are highly susceptible to invasive fungal diseases [2]. Posaconazole (PSCZ) is a triazole antifungal agent commonly used for the treatment and prophylaxis of invasive fungal infections [3] and functions as a potent inhibitor of cytochrome P450 (CYP) 3A4 [4]. Therefore, clinically significant drug–drug interactions may occur when PSCZ is co-administered with CYP3A substrates.

TAC is primarily metabolized by CYP3A4/5 and is a substrate of P-glycoprotein (P-gp). Consequently, the concomitant administration of PSCZ markedly increases TAC blood concentration in solid organ transplant patients, and various strategies for managing TAC concentration during PSCZ therapy have been extensively investigated [5, 6]. In these reports, the TAC trough concentration/dose (C/D) ratio elevated to 4.4-fold on average, and TAC dose reduction to approximately one-third has been suggested to maintain TAC therapeutic ranges. EVL is predominantly metabolized by CYP3A enzymes and is eliminated mainly via biliary excretion. In addition, EVL is also a substrate of P-gp [7]. Despite these pharmacokinetic similarities, quantitative clinical data regarding drug–drug interactions between EVL and PSCZ remain limited compared to those between TAC and PSCZ. Several studies have reported the interactions between EVL and other triazole antifungal agents with CYP3A-inhibitory properties. For example, with voriconazole, the mean C/D ratio of EVL increased approximately 8.7-fold during cotreatment therapy in lung transplant recipients, and EVL trough concentrations increased 7.5-fold in renal transplant recipients [8, 9]. Similarly, co-administration of itraconazole was associated with a 74% reduction in EVL clearance in renal transplant recipients [10]. A previous study demonstrated that the EVL trough C/D ratio was 3.5-fold higher during PSCZ co-administration than that without PSCZ in lung transplant recipients [11]. Another study revealed that a 3.8-fold increase in EVL trough concentration during PSCZ therapy in a renal transplant recipient [9]. Based on these previous reports, the drug–drug interaction between EVL and PSCZ might be expected to be of comparable magnitude to that between TAC and PSCZ. However, we observed a marked elevation in the EVL concentration induced by PSCZ in a liver transplant case.

The recommended EVL trough concentration range is 3–8 ng/mL for kidney, liver, and heart transplantation patients [12]. EVL trough concentrations exceeding 8 ng/mL have been associated with a higher incidence of adverse events, such as thrombocytopenia and hypertriglyceridemia, than concentrations within the 3–8 ng/mL range (thrombocytopenia: 3–9%, hypertriglyceridemia: 22–39%, respectively) [13]. Furthermore, Shihab et al. evaluated EVL-related adverse events across trough concentration categories (< 3, 3–6, 6–8, 8–12, and > 12 ng/mL) and reported that the lowest incidence of adverse events, including delayed wound healing, peripheral edema, dyslipidemia, and proteinuria, was observed in the 3–8 ng/mL group [14]. Therefore, maintaining blood concentrations of EVL within the therapeutic range is essential for minimizing exposure-dependent toxicities.

Here, we report the case of a liver transplant patient who received concomitant PSCZ and EVL therapy for allergic bronchopulmonary mycosis, resulting in a marked elevation of EVL blood concentrations. This report describes the clinically observed drug–drug interactions between PSCZ and EVL in a liver transplant case and discusses practical management strategies in transplant settings.

## Case presentation

The patient was a woman in her sixties who had undergone living-donor liver transplantation at our institution, Kyoto University Hospital, for hepatitis B virus-induced cirrhosis and hepatorenal syndrome 8 years prior to 20XX (20XX-8). Table 1 represents the patient characteristics, baseline laboratory parameters, and prescribed medications. The patient was prescribed with EVL (Certican®) 1.5 mg/day and extended-release TAC (Graceptor®) 0.2 mg once every two days. The trough EVL level was well controlled at approximately 5 ng/mL (target trough value: approximately 5 ng/mL), and the trough TAC level was also well controlled at approximately 0.5 ng/mL (target trough value: 0.5–2 ng/mL). Her regular medications included EVL (0.75 mg twice daily), TAC (0.2 mg once every two days), mycophenolate mofetil (250 mg twice daily), prednisolone (1.5 mg once daily), and entecavir (0.5 mg once every two days). The genetic polymorphism of *CYP3A5* in this patient was *3/*3. At our institution, *CYP3A5* genetic polymorphism is routinely assessed in liver transplant recipients receiving TAC, provided that informed consent is obtained, because it is known to significantly influence TAC pharmacokinetics and support individualized dose optimization.

**Table 1:** Baseline characteristics, laboratory parameters, and medications and doses administered

Variable	
**Characteristics**	
Age (years)	60s
Sex	Female
Height (cm)	151.8
Weight (kg)	46.9
*CYP3A5* genotype	*3/*3
**Laboratory parameters**	
Aspartate aminotransferase (U/L)	22
Alanine aminotransferase (U/L)	17
Total bilirubin (mg/dL)	0.5
Albumin (g/dL)	4.2
Prothrombin time-international normalized ratio (PT-INR)	1.01
Platelet (×10^9^/L)	157
Creatinine (mg/dL)	0.81
Estimated glomerular filtration rate (eGFR) (mL/min/1.73 m^2^)	54.2
Potassium (mmol/L)	4.7
Type IV collagen 7 S (ng/mL)	3.8
Autotaxin (mg/L)	1.034
Mac2-binding protein glycosylation isomer (M2BPGi, cut-off index)	0.57
**Medications**	
Everolimus	1.5 mg/day
Tacrolimus (extended-release)	0.2 mg every two days
Mycophenolate mofetil	500 mg/day
Prednisolone	1.5 mg/day
Entecavir	0.5 mg every two days

**Fig. 1:**
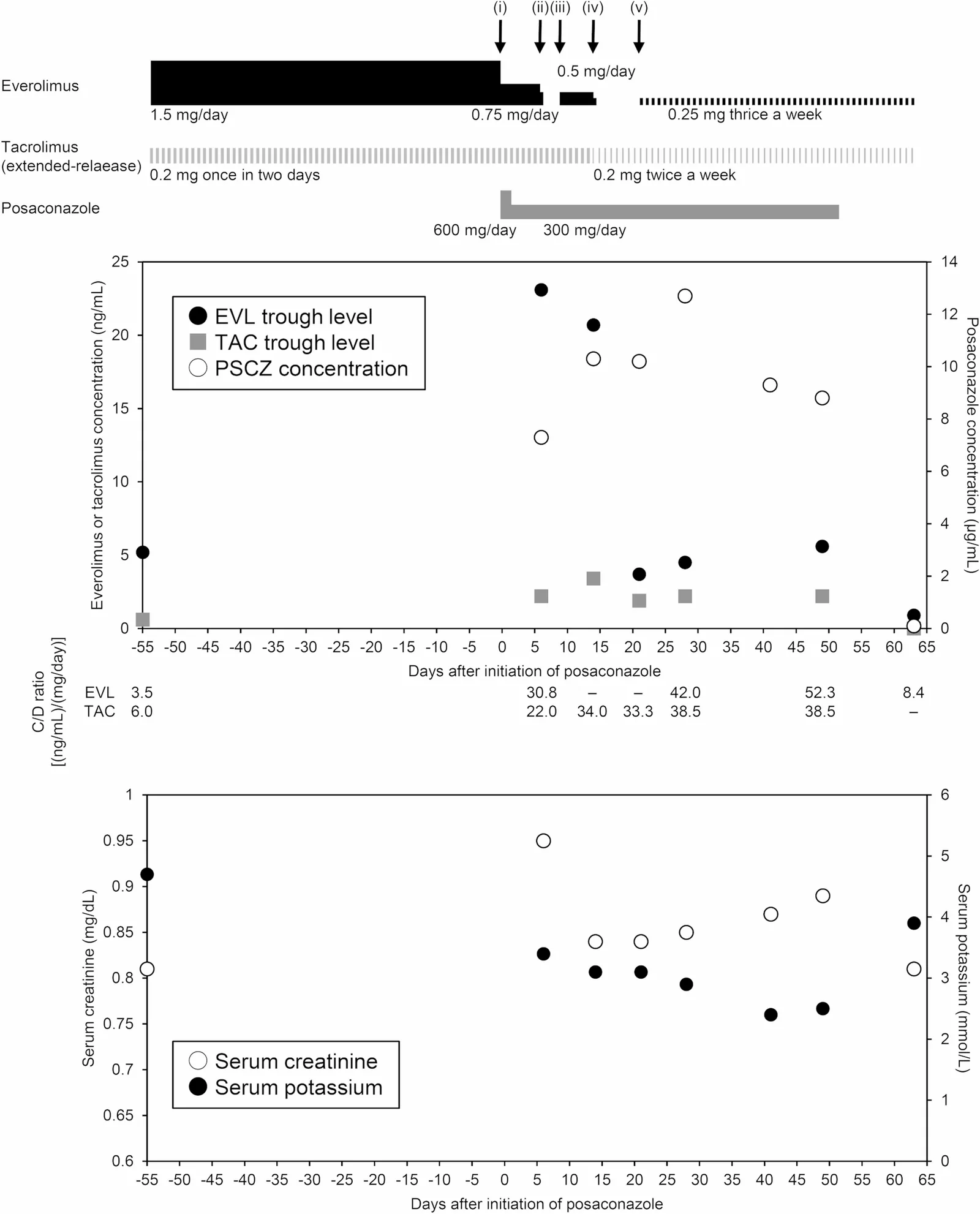
The clinical course of the patient. EVL, TAC, and PSCZ doses, EVL and TAC trough levels, PSCZ concentrations, and laboratory parameters are shown. The C/D ratio was calculated as the drug concentration divided by the average daily dose over the last 7 days. For the C/D ratio, the daily dose was defined as the total dose administered over 7 days divided by 7. If a drug-free period was included, the daily dose could not be calculated and was denoted by a hyphen (–). If the concentration was below the limit of detection, the C/D ratio could not be calculated and was denoted by a hyphen (–). Panels (i) – (v) correspond to the intervention points described in the text. EVL, everolimus; TAC, tacrolimus; PSCZ, posaconazole; C/D, concentration/dose

The patient experienced cough and was treated by inhaled corticosteroid/long-acting β-agonists (mometasone furoate 80 µg/indacaterol 150 µg once daily) and carbocisteine (500 mg twice daily), but symptoms persisted. A chest computed tomography scan revealed residual opacity and sputum culture detected filamentous fungi. The patient was suspected to have allergic bronchopulmonary mycosis. The patient’s primary surgeon and respiratory physician discussed the treatment, and oral PSCZ was prescribed (300 mg twice daily on day 0, followed by 300 mg once daily from day 1). Figure 1 summarizes the clinical course, including doses, TAC/EVL/PSCZ blood concentration levels, and serum creatinine and potassium levels. Considering possible drug–drug interactions, EVL was reduced to a half dosage (0.5 mg in the morning and 0.25 mg in the evening), and TAC was continued with the same dose (Fig. 1 (i)). The trough EVL and TAC levels 55 days prior to the start of PSCZ (day − 55) were 5.2 ng/mL and 0.6 ng/mL, respectively. Given the low baseline TAC concentration, the target levels were re-evaluated in consultation with the transplant physician, and a higher target concentration (up to approximately 2 ng/mL) was considered appropriate because TAC blood concentration of 0.6 ng/mL on day − 55 was low, and this fluctuation was within acceptable ranges. Accordingly, the TAC dose was maintained at the same dose. On day 6, the trough EVL level was high at 23.1 ng/mL and the trough TAC level was 2.2 ng/mL, but the patient did not develop any apparent adverse effects such as stomatitis or pancytopenia. In addition, the trough concentration of PSCZ was 7.3 µg/mL. McCreary et al. reported that toxicity threshold for PSCZ trough levels was above 3–3.75 µg/mL [15]; the trough PSCZ level was above the target range at the time of measurement. In Japan, therapeutic drug monitoring of PSCZ is not routinely performed in clinical practice, partly because it is not covered by the national health insurance system. Therefore, dose adjustment based on serum concentrations was not undertaken in this case, and treatment decisions were guided primarily by clinical assessment and tolerability.

The patient’s serum potassium level decreased from 4.7 mmol/L (day − 55) to 3.4 mmol/L, which was attributed to an adverse effect of PSCZ administration. EVL was discontinued from the evening of day 6 up to day 8 (Fig. 1 (ii)) and resumed at 0.5 mg/day on day 9 (Fig. 1 (iii)). TAC and PSCZ were administered at the same doses. On day 14, the trough EVL level remained high at 20.7 ng/mL, the TAC level was 3.4 ng/mL, and the PSCZ level was 10.3 µg/mL. On the same day, the platelet count was 105 × 10^9^/L, showing a decreasing trend (Table 2). Drug administration was suspended from the evening of day 6 to that of day 8 and then restarted at 0.5 mg/day. The subsequent blood EVL concentration was 20.7 ng/mL. Since this was approximately 4-times higher than the target trough concentration, reducing the dose by at least one quarter was necessary. Given the possibility that CYP3A inhibitory effects might be further enhanced after starting PSCZ, and that the next outpatient visit was scheduled after 1 week, discontinuation of the drug from the evening of day 14 to day 20 was decided.

**Table 2:** Results of laboratory tests regarding liver functions

Laboratory parameters	Day − 55	Day 6	Day 14	Day 21	Day 63
Aspartate aminotransferase (U/L)	22	20	18	19	17
Alanine aminotransferase (U/L)	17	15	13	14	13
Total bilirubin (mg/dL)	0.5	0.5	0.6	0.5	0.7
Albumin (g/dL)	4.2	3.8	3.3	3.3	3.4
Prothrombin time-international normalized ratio (PT-INR)	1.01	0.99	1.03	1.08	1.08
Platelet (×10^9^/L)	157	151	105	121	182
Type IV collagen 7 S (ng/mL)	3.8	–	–	–	5.2
Autotaxin (mg/L)	1.034	–	–	–	0.881
Mac-2-binding protein glycosylation isomer (M2BPGi, cut-off index)	0.57	–	–	–	1.2

EVL was again discontinued from the evening of day 14 to that of day 20 (Fig. 1 (iv)). During this period, TAC was reduced to 0.2 mg twice per week, while PSCZ was continued at the same dose. On day 21, the trough EVL level was 3.7 ng/mL, which was within the target range. The TAC level was 1.9 ng/mL, which was within the target range. EVL was resumed at 0.25 mg thrice per week, and TAC and PSCZ were continued at the same dose (Fig. 1 (v)). EVL was administered on Tuesdays, Thursdays, and Saturdays, and TAC was administered on Tuesdays and Fridays. Because EVL and TAC were administered following different dosing schedules, the days of a week were specified to facilitate medication adherence. The patient’s adherence was consistently maintained. On day 28, the patient experienced leg edema with an increase in body weight of 2 kg. On day 41, her serum potassium level decreased to 2.4 mmol/L, and potassium chloride tablets (600 mg twice daily) were started. On day 49, her serum potassium level was 2.5 mmol/L, and the trough PSCZ level was 8.8 µg/mL. Her lung opacities improved, and PSCZ treatment was completed. Even after that, EVL (0.25 mg thrice a week) and TAC (0.2 mg twice a week) administration were continued at the same doses. Post-transplant conditions of the patient were stable, and immunosuppressive agents comprised four medications (TAC, EVL, mycophenolate mofetil, and prednisolone); therefore, urgent increases of TAC and EVL doses were considered unnecessary. On day 63, her serum potassium level increased to 3.9 mmol/L, leg edema improved, and lung opacity did not worsen. Although the EVL and TAC trough levels decreased to 0.9 ng/mL and < 0.5 ng/mL respectively, her transaminases and bilirubin levels remained within the normal ranges (Table 2).

### Discussion and conclusions

Administration of immunosuppressants after liver transplantation is necessary to prevent rejection. Because immunosuppressants generally have narrow therapeutic windows, careful attention must be paid to potential drug–drug interactions, particularly in transplant recipients who frequently require concomitant medications. In the present case, marked increases in the C/D ratios of EVL and TAC of approximately 15-fold and 6-fold, respectively, were observed during PSCZ therapy.

PSCZ is a potent inhibitor of CYP3A [4], and several studies have demonstrated that PSCZ significantly increases TAC exposure in patients undergoing solid organ transplantation [5, 6, 11]. In contrast, clinical data regarding drug–drug interactions between EVL and PSCZ remain limited. Chanoine et al. reported that the EVL trough C/D ratio was 3.5-fold higher during PSCZ co-administration than that without PSCZ in lung transplant recipients [11]. Similarly, Billaud et al. described a 3.8-fold increase in EVL trough concentration during PSCZ therapy in a renal transplant recipient [9]. However, to the best of our knowledge, no previous studies have described this interaction in liver transplant recipients. In the present report, we describe a liver transplant recipient in whom the concomitant administration of EVL and PSCZ resulted in a markedly greater increase in EVL exposure than previously reported. Although the liver function test results were almost within the normal range, given slightly elevated levels of type IV collagen and Mac-2-binding protein glycosylation isomer (M2BPGi), which are the markers of hepatic fibrosis, certain abnormalities in liver function after liver transplantation may have influenced EVL and/or PSCZ metabolism.

To explore the mechanism underlying this pronounced interaction, we applied the contribution ratio of the target metabolizing enzyme to the oral clearance of substrates and inhibition ratio of inhibitors (CR–IR method), which predicts the magnitude of drug–drug interactions based on the contribution of a metabolizing enzyme to the oral clearance of a substrate and the inhibitory potency of a co-administered drug [16]. Although systemic exposure is ideally assessed using the area under the concentration–time curve (AUC), its measurement is often impractical in routine clinical settings. Therefore, the C/D ratio was used as a surrogate marker in this study; however, it does not directly correspond to AUC and should be interpreted cautiously. In the CR–IR method, the contribution of CYP3A to oral clearance (CR_CYP3A_) and the inhibition ratio of CYP3A (IR_CYP3A_) were used to estimate the increase in the AUC according to the following equation:

$${\rm{AUC}}\,{\rm{increase = 1/(1 - C}}{{\rm{R}}_{{\rm{CYP3A}}}} \times {\rm{I}}{{\rm{R}}_{{\rm{CYP3A}}}}{\rm{)}}.$$

Based on previous reports, CR_CYP3A_ values for EVL and TAC were estimated to be 0.95 and 0.66, respectively, and the IR_CYP3A_ value for PSCZ was estimated to be 0.87 [7, 16–18]. Using these parameters, the predicted AUC increases for EVL and TAC during PSCZ co-administration were approximately 5.8-fold and 2.3-fold, respectively.

In contrast, in the present case, the observed increases in the C/D ratios of EVL and TAC after 49 days of PSCZ therapy were approximately 15-fold and 6.4-fold, respectively, which substantially exceeded the predicted values. The trough concentration of PSCZ reached 8.8 µg/mL, which was markedly higher than previously reported levels. McCreary et al. reported that the target PSCZ trough concentrations are ≥ 0.5–0.7 µg/mL for prophylaxis and ≥ 1–1.5 µg/mL for treatment, and trough concentrations exceeding 3–3.75 µg/mL are associated with an increased risk of toxicity [15]. The observed concentration in this case substantially exceeded these thresholds. Relatively small body size and advanced age of the patient may have contributed to the elevated PSCZ exposure, as discussed later. Accordingly, this case may be more appropriately interpreted as a drug–drug interaction occurring under conditions of excessive PSCZ exposure, rather than typical co-administration. Such a high PSCZ concentration may have resulted in near-complete inhibition of CYP3A activity, thereby enhancing its interaction with EVL. Indeed, previous studies have demonstrated that high PSCZ concentrations significantly increase the mean trough levels of midazolam, a prototypical CYP3A substrate [17, 18]. In addition, co-administration of EVL and ketoconazole, a potent CYP3A inhibitor, increased EVL exposure by 11.2–17-fold [19]. Assuming an IR_CYP3A_ value of 0.99, the predicted AUC for EVL and TAC would be approximately 16.8-fold and 2.9-fold, respectively. This estimate closely approximates the observed increase in EVL exposure in the present case, although a discrepancy remains for TAC. As this is a single case report, the findings may not be generalizable to other patients. The PSCZ concentration in this case was markedly elevated compared with typical clinical settings, which may limit the applicability to other patients. Furthermore, the mechanistic interpretation based on the CR–IR method should be considered a plausible explanation, and other potential mechanisms cannot be excluded.

The greater increase in EVL exposure compared with TAC may be primarily explained by the higher dependence of EVL on CYP3A-mediated metabolism. In contrast, *CYP3A5* polymorphism may have contributed to interindividual variability in TAC exposure as a secondary factor. Notably, the patient harbored the CYP3A5*3/*3 genotype, which is associated with the absence of CYP3A5 metabolic activity. This genetic background may further amplify the dependence of drug clearance on CYP3A4, potentially contributing to the unexpectedly large increase in EVL exposure. More detailed pharmacogenetic and pharmacokinetic analyses are required to fully elucidate the observed discrepancies, particularly for TAC.

The PSCZ trough concentrations in this study were consistent with the reported toxicity ranges. Several population pharmacokinetic studies identified factors associated with elevated PSCZ exposure. Kohl et al. reported that increasing age is associated with a decreased volume of distribution [20], whereas Vehreschild et al. demonstrated that higher body weight is correlated with a larger apparent volume of distribution [21]. In addition, AbuTarif et al. identified diarrhea, proton pump inhibitor use, and elevated serum bilirubin or gamma-glutamyl transferase levels as significant covariates affecting PSCZ exposure [22]. In the present case, there were no problematic issues with the patient’s medication adherence or food intake, and no diarrhea was observed. The patient was in her sixties and had a body weight of 46.9 kg, with a BMI of 20.3, which was not indicative of underweight status but reflected a relatively small body size. Together, these findings suggest that patient-specific factors, including advanced age and relatively small body size, may have contributed to the elevated PSCZ concentration observed in this case. Although liver enzyme levels remained within normal ranges throughout the observation period, mild changes in synthetic function markers, such as albumin and platelet counts, and slight elevation of type IV collagen and M2BPGi levels were observed (Tables 1 and 2), suggesting that liver function may not have been entirely stable. These factors may also have influenced PSCZ exposure to some extent.

Hypokalemia has been reported as an adverse effect of PSCZ [23–25]. The underlying mechanism is thought to involve PSCZ-induced pseudohyperaldosteronism (PIPH), which is characterized by secondary hypertension, hypokalemia, and occasional metabolic alkalosis [26]. PIPH has been shown to be associated with elevated PSCZ blood concentrations [27]. Although renin–aldosterone levels were not measured, the temporal association with high PSCZ exposure suggests possible PIPH. Furthermore, in the present case, hypokalemia and leg edema developed concomitantly with high PSCZ exposure, suggesting that PIPH may have contributed to these symptoms. Hypokalemia due to PIPH is unlikely to be directly related to EVL, and is a recognized adverse effect associated with PSCZ.

Serum creatinine showed a slight increase from day 14 to day 49; however, the magnitude of change was small and therefore it was not considered clinically significant. A clear causal relationship with EVL, TAC, or PSCZ could not be established. This mild fluctuation may reflect variability related to clinical conditions or concomitant medications.

In conclusion, liver transplant recipients receiving long-term immunosuppressive therapy with CNI and mTOR inhibitors are at a high risk of clinically significant drug–drug interactions when triazole antifungal agents are prescribed. Our findings suggest that the interaction between EVL and PSCZ may be more pronounced than that between TAC and PSCZ, particularly in patients with high PSCZ exposure. When PSCZ is administered, especially to elderly or underweight patients, clinicians should be vigilant not only for PSCZ-related adverse effects, such as hypokalemia due to PIPH, but also for unexpectedly strong pharmacokinetic interactions with EVL. Therapeutic drug monitoring of immunosuppressants is essential, whereas monitoring of PSCZ concentrations may be considered in selected cases to optimize safety and avoid excessive exposure, particularly when adverse events are suspected or clinically significant drug–drug interactions are anticipated. However, as measurement of PSCZ concentrations is not covered by insurance, routine monitoring may be challenging in some clinical settings and should therefore be reserved for selected clinical scenarios.

## Data Availability

No datasets were generated or analysed during the current study.

## Abbreviations

AUC: Area under the concentration-time curve
C/D: Concentration/dose
CNIs: Calcineurin inhibitors
CR: Contribution ratio
CYP: Cytochrome P450
EVL: Everolimus
IR: Inhibition ratio
mTOR: Mammalian target of rapamycin
P-gp: P-glycoprotein
PIPH: Posaconazole-induced pseudohyperaldosteronism
PPI: Proton pump inhibitor
PSCZ: Posaconazole
TAC: Tacrolimus
